# Effectiveness of community mental health nurses in an integrated primary care service: An observational cohort study

**DOI:** 10.1016/j.ijnsa.2024.100182

**Published:** 2024-02-05

**Authors:** Mark Kenwright, Paula Fairclough, Jason McDonald, Louisa Pickford

**Affiliations:** a University of Staffordshire, Centre for Health Innovation, Blackheath Lane, Stafford, ST18 0YB, England; b Keele University, The Darwin Building, Keele, Staffordshire, ST5 5BG, England; c Combined Healthcare NHS Trust, Wellbeing Service, Lancaster Buildings, High Street, Newcastle-under-Lyme, ST5 1RH, England

**Keywords:** Community mental health nurses, Effectiveness, Integrated primary care teams, Improving access to psychological therapies, Co-morbidities

## Abstract

**Background:**

The movement of community mental health nurses into primary care is important for the delivery of primary care integrated teams. There is little evidence or guidance on how integration should be implemented, or on the effectiveness of mental health nurses in primary care.

**Objectives:**

1. Examine one method of integrating community mental health nurses in a primary care mental health service to identify factors that both facilitated and hindered integration. 2. Report on the outcomes of community mental health nurses in delivering problem-specific evidence-based psychological interventions in primary care.

**Design:**

A naturalistic observational cohort study

**Setting:**

An integrated primary care mental health service in the UK North Midlands

**Participants:**

1,582 referrals from 1st April 2019 – 31st March 2022.

**Method:**

Anonymised patient records from routine treatment with community mental health nurses in an integrated primary care service were extracted and analysed to identify patient characteristics, content of treatment and outcomes. Features of service design were also examined to report on aids and barriers to primary care integration.

**Results:**

Large and clinically significant pre to post treatment effect sizes of between 0.5 and 0.8 were observed in symptom reduction and functional improvement for patients treated by community mental health nurses for a range of mental health problems. Aids to integration were: A single line of clinical management and governance; shared training across all roles; a shared IT system/electronic appointment diary. Barriers to integration were: Different contract management structures, and different clinical IT systems across primary and secondary care.

**Conclusions:**

Integrating community mental health nurses into one primary care mental health service comprising different mental health professionals provided a single point of access to different mental health treatments. Primary care community mental health nurses delivered effective evidence-based psychological interventions in a stepped-care model that reduced demands on secondary care services.

**What is already known**

• Current healthcare strategy is to integrate community mental health nurses into multidisciplinary primary care teams, but different providers and lines of accountability can be a barrier to this in primary care.
• There is little evidence-based guidance on how primary care integration should be implemented.
• There is a lack of consistent evidence for the effectiveness of community mental health nurses in primary care.

Alt-text: Unlabelled box

**What this paper adds**

• This study describes how community mental health nurses were effectively integrated into a single primary care mental health service, working alongside physical healthcare practitioners.
• The service delivered a convenient, single point of access for mental health problems that reduced both transitions between teams and demands on secondary care.
• Community mental health nurses delivered evidence-based, problem-focused, psychological interventions in a stepped-care model that significantly improved patient symptoms and functioning.

Alt-text: Unlabelled box

## 1 Introduction

The UK National Health Service is implementing a strategy to move community mental health teams away from the traditional secondary care service model, to work within new multidisciplinary primary care teams based around doctor's practices (General Practitioners) that serve smaller neighbourhood populations (NHS England, 2019). The strategy is part of a Long-Term Plan to deliver integrated care for the physical and mental health needs of patients in primary care community settings (NHS England, 2019).

A review of the current evidence base reveals two challenges facing community mental health nursing in delivering this strategy – 1) The lack of guidance/evidence on how to best implement integration of mental health services in primary care; 2) The lack of evidence on both treatment content and effectiveness of community mental health nursing practice.

The lack of guidance on how to best integrate mental health services is compounded in the UK by a move to ‘Practice-based commissioning’ (Dept of Health, 2005) where primary care physicians now commission services for their own practices from a range of different providers. As a result of this, three different mental health worker roles now compete for provision in primary care:

1) GP practices directly commission/employ primary care mental health nurses to work in their offices/consultation rooms to treat less severe mental health problems.
2) GPs also commission psychological therapists/practitioners from the ‘Improving access to psychological therapies’ programme to treat people with anxiety disorders and/or depression (common mental health problems).
3) NHS mental health trusts also provide community mental health nurses to work with specific GP practices. These nurses have traditionally worked as part of the secondary care psychiatrist-led community mental health teams to treat more severe and complex problems.

Although integration generally leads to higher satisfaction for both primary care staff and patients (Ede et al., 2015), the lack of guidance on how different mental health service providers can best integrate may allow some of the barriers to effective integration to continue. These include: Lack of staff knowledge about system structures and work processes; lack of integrated health professionals’ timetables; uncoordinated care planning; no clearly defined integrated clinic roles and disjointed services within a decentralised system (Wakida et al., 2018).

The UK government-funded Improving Access to Psychological Therapies (IAPT) programme was introduced in 2008, and has since trained over 10,500 psychological therapists to treat over a million patients a year with common mental health problems (NHS England, 2018). Therapists work alongside other health professionals in integrated primary care teams, and use sessional outcome measures to demonstrate that over half of patients move to recovery on measures of both anxiety and depression (NHS Digital, 2022). This successful integration of routine data collection to demonstrate effectiveness creates issues for the community mental health nurses who work alongside these psychological therapists, as conversely, there are few studies demonstrating the effectiveness of community mental health nurses in primary care.

Qualitative evidence shows that GPs value the role of community mental health nurses working in primary care liaison roles (McLeod and Simpson, 2017), and when mental health nurses deliver specific evidence-based psychological interventions for defined mental health problems, such as Cognitive Behavioural Therapy (CBT) for psychosis, patients show significant symptom improvement (Malik et al., 2009). Yet when they deliver generic interventions across all problems (without adherence to the evidence-base for each), there have been no demonstrable improvements over usual GP care (Kendrick et al., 2006: Veen et al., 2021), or on subsequent mental health admission rates (Leach et al., 2020). A similar failure of generic interventions is also reported by primary care mental health workers outside of nursing (Woods et al., 2020).

This inconsistent evidence base for the effectiveness of community mental health nurses may be due in part to the wide variety of interventions provided for such a wide range of problems, resulting in a lack of clarity/focus for research. Systematic reviews of the role indicate that the content of care delivered by these nurses differs greatly between services/settings, with “Care co-ordination” and “Case management” the most commonly recorded intervention (Kudless and White, 2007; Heslop et al., 2016; Kidd et al., 2017). Average caseloads range between 35 - 45 patients - well above the recommended range of 10–30 (Happell et al., 2012), and the amount of clinical contact per patient varies between 2 sessions over a couple of weeks to 40 plus sessions over 2 years (Kidd et al., 2017).

Therefore, there is a need to examine and better define the interventions delivered by community mental health nurses, such as the proportion of time they spend delivering psychotherapeutic or psychological interventions, and their effectiveness.

This study contributes to the field by describing the implementation of an integrated primary care mental health service in the UK's North Midlands region. An evaluation of the service aims to provide evidence to inform and clarify two aspects of community mental health nursing practice –

1. A model of primary care mental health integration is presented and examined to identify both the successful components enabling the achievement of seamless care with a single assessment, as well as the barriers to effective integration for community mental health nurses.
2. The working practices and interventions delivered by community mental health nurses in primary care are examined, to identify the key features of a recovery-focused, stepped-care model of mental health nursing, along with outcomes to evaluate service performance.

## 2 Methods

This was a naturalistic, observational cohort study carried out in a medium sized primary care mental health service in the UK North Midlands region. Anonymised data was extracted for 1582 patients who entered treatment with CMHNs between 1st April 2019, and 31st March 2022 (with a cut-off for discharge and data extraction of 31st September 2022). All patients routinely provided informed consent for their data to be recorded and used in an anonymised form for audit and research purposes.

### 2.1 The service

The service was designed from the patient's perspective through feedback gained in service user meetings. Nurses routinely invited all service users to join a monthly “Expert advisory group”. Refreshments and travel expenses were provided, and a changing group of between 4 −10 service users volunteered to attend each meeting. The meetings were led by senior mental health nurses who introduced/presented various aspects of the service, facilitated service users to share their views on delivery and wrote the groups suggestions/views on a whiteboard. Suggestions were written-up and fed into the monthly service steering group attended by senior nurses and managers. The resulting formal service procedures and clinical protocols were presented in subsequent service user meetings for corrections/amendments. This led to a co-produced model of integrated care where people with any mental health problem could easily book an appointment, tell their story once and start an evidence-based treatment in a convenient primary care “Wellbeing Service”.

The Wellbeing Service was provided as a partnership between two NHS trusts and three voluntary sector organisations. Originally a stand-alone ‘Improving access to psychological therapies’ service employing mainly CBT Therapists to treat common mental health problems, the service was developed further by integrating community mental health nurses from secondary care community mental health teams to work alongside these therapists in one multidisciplinary team. All mental health professionals were allocated to serve specific GP practices, ensuring that each practice received the equivalent combination of capacity from each profession. Each practice served a specific geographical area and population (ranging from around 4000 to 12,000 patients per practice).

Each GP practice team contained: GP partners; GP registrars; Community Matrons (Senior Nurses); Community Nurses (Physical health); Practice Nurses (Physical health), Occupational Therapists; Physiotherapists; Podiatrists; Pharmacists and Administrators. The community mental health nurses had allocated working times/days in each practice. Other professionals could book patients directly into the mental health nurses appointment diary on the clinical IT system, but they were also available at set times to discuss patients directly with practice staff. On joining each practice, the mental health nurses delivered training sessions to the practice staff on identifying mental health problems and referral protocols using illustrative case examples. They also attended regular practice team meetings led by the GP partners to discuss patients and team performance.

The mental health workers all adhered to the principles of a ‘Stepped-care’ model of treatment for people with any mental health problem, whose needs could be met by one mental health professional in primary care –

Step 1 – Comprises ‘watchful waiting’ by the GP, who may provide self-help information and continue to monitor patient's mental health over multiple appointments.
Step 2 - In the “Improving access to psychological therapies programme” patient with mild-moderate anxiety and depression were treated by Psychological Wellbeing Practitioners (typically psychology graduates with an additional 12 month's clinical training) who support patients to use guided self-help programmes.
Step 3 - Unresponsive patients with anxiety disorders and depression were ‘stepped-up’ to CBT Therapists or Counsellors who had post-graduate level training to specialise in delivering one type of therapy (CBT, Counselling or Interpersonal Therapy) for these disorders only. Patients with problems other than anxiety disorders and depression, such as: personality disorders; eating disorders; complex bereavement; complex trauma symptoms from abuse (childhood or recent e.g. domestic abuse); adjustment disorder; somatic symptom disorder and anger management were all treated by the community mental health nurses in primary care.
Step 4 - Patients whose problems were of a severity/complexity requiring input from multiple mental health professionals (e.g. specialist medication and/or social care needs) were ‘stepped-up’ to the psychiatrist-led community mental health team by the community mental health nurses through attendance at a weekly “Multidisciplinary Team Meeting” in which cases were presented and handed over. Patients could then be treated by a range of professionals, including Clinical Psychologists, who specialise in treating more complex/severe problems. This ensured a seamless “Step-up” to the care of this psychiatrist-led mental health team.

All the mental health professionals had access to the same secure online clinical IT system to manage patient notes, appointments and outcome data. This included shared appointment diaries for assessments and first treatment sessions. Each mental health professional allocated protected appointments in their diary each week for first appointments that could be booked into by other workers. This was a crucial design feature of the service, ensuring a single point of access for all mental health problems in which patients have only one assessment regardless of their problem/treatment. This aimed to improve efficiency and lower waiting times but requiring trust and shared understanding across different mental health professional roles.

### 2.2 The workforce

The service comprised 71 Whole Time Equivalent clinical posts, and 5 administrative/managerial posts. Between 6 to 21 trainees were also employed and supervised within the service at any point in time during the study period.

### 2.3 Community mental health nurses (Wellbeing nurses)

10 community mental health nurses moved into the primary care mental health service (The Wellbeing Service) from two secondary care community mental health teams. The outcomes of these nurses are the focus of this study, and are presented in the results section. On moving into the primary care Wellbeing Service the nurses received a 3-day programme of additional training on how to assess and allocate different problems to the various mental health professionals across the team. All nurses were allocated to staff the service ‘Referral line’ on a roster, dealing with self-referral telephone calls/web-referrals, as well as professional referrals. The nurses carried out initial screening of all calls to provisionally identify patient's problems and offer them the next available first appointment with the most suitable mental health professional. This was made possible through the use of a shared electronic appointment diary for first appointments.

At the first appointment, which lasted an hour, the community mental health nurses carried out a problem-focused assessment to identify each patient's main problem, and which appropriate evidence-based treatment to offer in line with guidance from the UK's National Institute for Health and Care Excellence (NICE). As this was not a full diagnostic structured clinical interview, the term “problem descriptor” was used in patient records rather than “diagnosis”.

If the outcome decision from this first appointment was that treatment from another mental health professional was required, the community mental health nurses booked patients into the next available first treatment appointment with the appropriate professional using the shared electronic appointment diary (although most first appointments booked with nurses were suitable as this was usually identified at the initial screening).

Community mental health nurses had a service target to spend over 50% of their working time delivering direct care to patients. This was supported by the clinical IT system, which provided a detailed report of clinical time by day/week/month. Nurses offered weekly treatment appointments of one hour duration in GP practices, primary care clinics, patient's homes, via web-camera or telephone. Appointments were structured, starting with the completion/review of outcome measures and agenda setting, moving on to completion of collaboratively agreed treatment interventions/tasks and ending with goal setting for the next appointment. Nurses delivered a range of evidence-based psychological interventions in a recovery-focused model of care, and initially scheduled 6 – 10 appointments. A collaborative review of progress/goals with patients informed the need for the next appointment. Some patients with longer-term, more severe problems went on to receive further appointments, scheduled with less frequency, for up to 12 months of support and monitoring. Average caseloads for the nurses were 28–30 patients.

These nurses completed a range of intensive training courses in evidence-based psychological therapies, some delivered within the service and others externally. Each nurse was trained in more than one, but not all therapies, which depended on professional interests and preferences. Therapies included: Supporting CBT guided self-help for chronic low self-esteem and low mood; Problem-solving; Behavioural Activation; Dialectical Behaviour Therapy for emotionally unstable personality disorder; CBT interventions for bipolar disorder and symptoms of psychosis; Behavioural Family Therapy; Solution-focused Brief Therapy; Motivational Interviewing and Person-centred Counselling.

### 2.4 Community mental health nurses employed solely in GP practices

8 community mental health nurses were commissioned directly by GP practices to work solely in their surgeries. These nurses worked differently to those in the Wellbeing Service, as they were managed by GP partners and practice managers to solely meet the needs of patients in their practices (as opposed to seeing patients in a flexible range of settings, such as primary care clinics and at home). When patients expressed a mental health problem, either on contacting the practice, or in a medical consultation, they were booked into a first appointment with the mental health nurse through the clinical IT system used by the practice. These appointments were delivered in the practice either face-to-face, by telephone or video-call.

These community mental health nurses spent a greater proportion of their time delivering assessments to meet the demands of busy GP practices. They therefore had less capacity to deliver treatment and referred more patients to other mental health professionals. This high volume of assessments released GP capacity that would have otherwise been taken up by patients with mental health problems. This was in contrast to the community mental health nurses in the Wellbeing Service, who spent a much greater proportion of their time delivering treatment.

Integration of these GP-practice-based community mental health nurses with the other community mental health nurses working in the Wellbeing Service was achieved by training from the Clinical Lead over two days on how to identify and allocate patients with different mental health problems to the appropriate professional across the three different primary care teams (1. GP Practice; 2. Wellbeing Service or 3. Community Mental Health Team). A critical aspect of this training was providing the GP practice mental health nurses with access to the same electronic appointment system as all other mental health professionals in the Wellbeing Service. This enabled GP practice nurses to book patients directly into first treatment appointments with any professional in the Wellbeing Service, and ensured that patients only required one assessment.

**Fig. 1:**
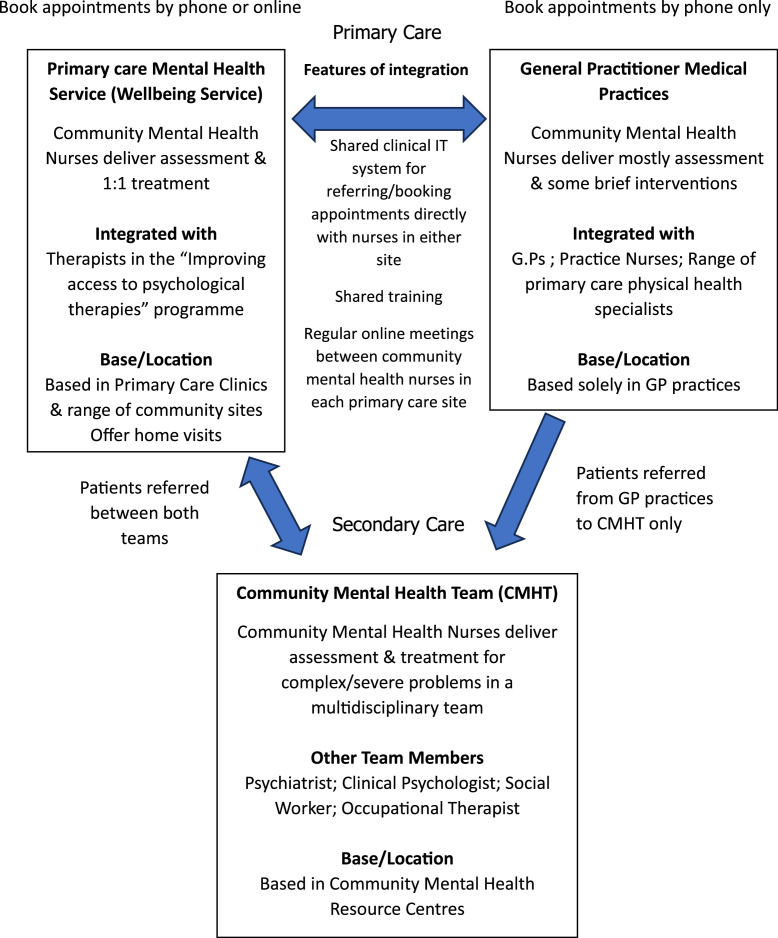
Flow chart showing the teams and locations of Community Mental Health Nurses.

### 2.5 Therapists in the primary care mental health service (Wellbeing service)

#### 2.5.1 Primary care counsellors

5 Primary Care Counsellors in the Wellbeing Service supported the community mental health nurses in providing both specialist bereavement counselling, and person-centred counselling for a wide range of problems. At a first appointment, nurses could book suitable patients directly into the next available first counselling appointment in the shared electronic diary. Counsellors offered appointments in a range of primary care sites, or via video-call, and initial treatment offer was 6–10 sessions, which could be extended on review (Fig. 1).

#### 2.5.2 Therapists in “The improving access to psychological therapies” (IAPT) programme

This section of the Wellbeing Service was the largest, comprising: 22 Cognitive Behavioural Therapists; 13 Psychological Wellbeing Practitioners; 5 Employment Advisors; 5 Counsellors/Interpersonal Therapists; 2 ‘Couples Therapists’; 4 Administrators and a Clinical Lead. These workers were trained and funded to specialise solely in the assessment and treatment of patients with specific anxiety disorders and depression (common mental health problems) in compliance with NICE guidelines and the IAPT programme. They did not treat any other problems. All the practitioners and therapists in the IAPT programme listed above used the same clinical appointment system as the community mental health nurses, so each mental health professional could book a patient into a first treatment appointment with any other professional in primary care, regardless of their role.

#### 2.5.3 The effect of the COVID-19 pandemic

It is important to acknowledge that the COVID pandemic drastically changed service delivery and patient need after the first year of this cohort study. In March 2020 the service moved entirely to remote delivery when the UK went into lockdown. All appointments were delivered remotely by telephone or web-camera until May 2021 when the service began to offer the choice of face-to-face appointments again in a blended delivery approach. All mental health professionals in the service retained the structure/length of treatment and appointments despite the change of delivery format. However, during the first few months of the lockdown many patients withdrew from treatment, referrals significantly reduced and the focus of treatment for many changed from achievement of specific recovery goals to support for mental health through the lockdown.

#### 2.5.4 Measures

Patients received an automated email 48 h prior to their appointment with a link to complete the self-report clinical measures online. The scores they entered were then displayed in their appointment record on the clinical IT system and could be reviewed by their nurse prior to the appointment. If patients had no internet access, or could not navigate the online measures, the community mental health nurse read out each item from the questionnaires at the start of the appointment and patients stated their score.

Wellbeing Nurses entered appointment data at each contact (including follow-ups), such as attendance, appointment format (face-to-face/video/telephone), purpose of appointment, interventions provided and duration of appointment.

The Wellbeing Nurses shared the same clinical data system as the psychological therapists, which was designed to collect and analyse scores on the clinical measures for depression, anxiety and general functioning at each clinical session. Whilst the nurses did not treat anxiety and depression as main problems, many patients also had symptoms of low mood, anxiety and reduced functioning, so nurses used these clinical measures at each treatment session, consisting of:

The Patient Health Questionnaire (PHQ-9) (Kroenke et al., 2001): a nine-item self-report questionnaire evaluating depressive symptomatology. Patients rate the frequency of symptoms over the previous 2 weeks e.g., “Little interest or pleasure in doing things: Not at all (0); Several days (1); More than half the days (2); Nearly every day (3)”. Total scores range from 0 (no symptoms) to 27 (severe depressive symptoms). Across 15 studies PHQ-9 scores > 10 had a sensitivity of 88% and a specificity of 88% for Major Depressive Disorder, with high internal consistency and reliability (Gilbody et al., 2007).

The Generalized Anxiety Disorder Questionnaire (GAD-7) (Spitzer et al., 2006): a seven-item self-report questionnaire focusing on symptoms of anxiety. Patients rate the frequency of symptoms over the previous 2 weeks e.g., “Feeling nervous, anxious or on edge: Not at all (0); Several days (1); More than half the days (2); Nearly every day (3)”. Total scores range from 0 (no anxiety symptoms) to 21 (severe anxiety symptoms). The GAD-7 has demonstrated good psychometric properties for diagnosing GAD, and high internal consistency and convergent validity in heterogeneous samples with mixed diagnoses (Johnson et al., 2019).

The Work and Social Adjustment Scale (WASA) (Mundt et al., 2002, a self-report questionnaire measuring the social and functional impact of a patient's main problem. The instrument is 5 questions long and assesses the impact of a person's mental health difficulties on their ability to function in terms of work, home management, social leisure, private leisure and personal or family relationships. Each item is rated on a 0–8 scale, and scores are sensitive to patient differences in disorder severity and treatment-related change. Cronbach's alpha measure of internal consistency for each item separately were above 0.7, demonstrating reliability across these subscales (Mundt et al., 2002).

#### 2.5.5 Statistical analysis

Paired *t*-tests were used to examine the mean pre-post treatment difference on each measure and p values. Effect sizes were calculated for the mean pre-post treatment change using the formula: pre-treatment mean – post-treatment mean)/pre-treatment SD.

## 3 Results

### 3.1 Patient characteristics

The Wellbeing Nurses treated 1419 patients beyond assessment between 1st April 2019, and 31st March 2022. Mean number of contacts per care episode was 9.3 (*SD 7.1; range 1–34)*, with a mean referral duration of 185 days (*SD 120; range 1–379).* This reflected the difference in need/input across patients, from those receiving short-term, frequent, intensive therapy sessions, to those requiring monitoring and support sessions spaced out over a longer-term period of up to 12 months.

**Fig. 2:**
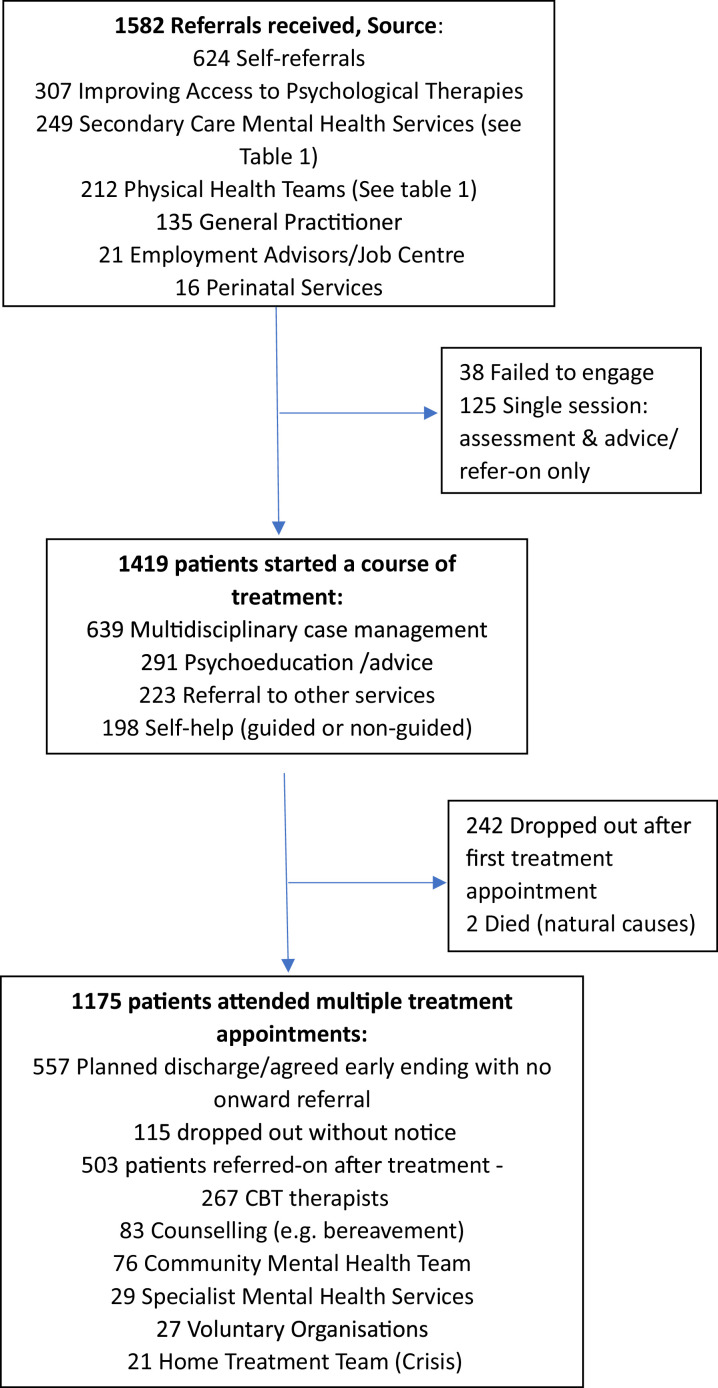
Flow diagram of patients through the Primary Care Community Mental Health Nursing Team.

90% of referrals were white British, which reflects a slightly greater ethnic diversity than the regional population in which the service is based, of which 94.1% is white (NHS, 2020). The majority of patients self-referred directly to the service (although many were advised to by GPs/Practice Nurses). Only 15% of referrals were ‘stepped down’ from secondary care mental health services (see Fig. 2). Referrals were lower in March-June 2020, as many people avoided NHS services during the pandemic restrictions. This slightly reduced the numbers treated during the study period (Table 1).

**Table 1:** Source of secondary care referrals to primary care community mental health nurses.

Mental Health Teams	
97	Community Mental Health Teams
46	Mental Health Access Team
40	Home Treatment Team (Crisis)
37	Liaison Psychiatry Team in Accident & Emergency Dept
18	Drug & Alcohol Team
11	Other (e.g. Parent & baby, Neuropsychiatry)

Physical Health Teams	
53	GP Practice Nurses
38	Community Respiratory
35	Cardio-vascular Teams
29	Diabetes
22	Pain Management & Physiotherapists
21	Community Nurse / Matron
14	Other (e.g. Cancer; Stroke)

13% of patients were referred directly by physical healthcare teams, which appears low considering that 37% of patients treated for mental health problems had a co-morbid long term physical health condition, and 17% had a registered disability. The high proportion of self-referrals that were directed by other primary care professionals may be a factor in these observed proportions (see Fig. 2 and Table 2).

**Table 2:** Characteristics of patients referred to primary care community mental health nurses.

Age (*N* = 1582 mean/SD)	37.2 (15.7)
Female/male (*N* = 1582)	1023 / 544
% White British (*N* = 1397)	90%
With a disability (*N* = 1494)	256 (17%)
Long term physical condition (*N* = 1537)	564 (37%)
Sexuality (*N* = 1255) : Heterosexual	1128 (90%)
Bisexual	52 (4%)
Homosexual	50 (4%)
Undecided / Attracted to neither gender	25 (2%)
Relationship status (*N* = 1338): Single	598 (45%)
Married	322 (24%)
Co-habiting	173 (13%)
Long-term relationship	146 (11%)
Divorced/separated	72 (5%)
Widow/Widower	27 (2%)
Risk identified (*N* = 1523) : No risk	225
Low risk	1172
Medium risk	118
High risk	8
Main problem recorded at assessment (*N* = 1544):	
Personality problems (emotional dysregulation)	319
Complex trauma (Past abuse)	235
Domestic /family abuse (current or recent)	222
Severe depression (pre-therapy stabilisation)	207
Severe anxiety disorder (pre-therapy stabilisation)	153
Eating disorder	70
Bereavement	65
Other or not recorded	64
Somatization disorder	49
Drug/alcohol abuse	44
Adjustment disorder	38
Anger management	32
Severe obsessions (over-valued ideas)	30
Bipolar depression (current symptoms mild)	16
Duration of main problem at assessment (*N* = 1366)	
1 - 3 months:	60
>3 - 6months:	78
>6 - 12 months:	222
>1 - 2 years:	421
>2 - 3 years:	371
>3 - 4 years:	153
>4 – 5 years:	9
>5 - 10 years:	17
>10 – 20 years:	26
>20 – 50 years:	9

50% of the main problems identified at assessment were either personality problems, complex trauma or abuse (see Table 2), with severe impact on relationship functioning on the WASA (52% of patients reporting their relationship status as either single or separated/divorced).

**Fig. 3:**
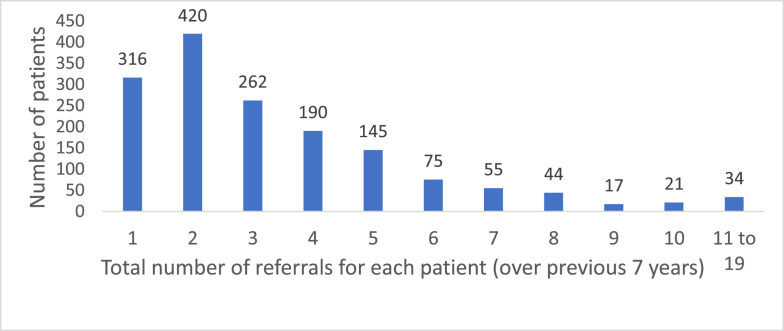
Patients grouped by total number of Community Mental Health Nurse referrals* (*N* = 1579).

77% of patients were categorised as “Low risk” (See Table 2), which meant they reported current/recent suicidal thoughts (or thoughts of harming others), but no current plans/significant history of past attempts, and protective factors in place. 73% reported a problem duration longer than 12 months (see Table 2). The majority of patients (56%) were allocated to mental health care clusters 4 (Non-Psychotic - Severe) or 5 (Non-Psychotic - Very Severe) at assessment (Fig. 3).

*Previous care episodes not restricted to within the study period

### 3.2 Characteristics of treatment delivery

The nurses delivered a range of structured, recovery-focused psychological interventions (see Table 3), matched to patient's main problems in line with evidence-based guidance (e.g. NICE) and according to the specific training courses completed through their professional development. The clinical database provided longitudinal data on the total number of care episodes each patient had received from the Wellbeing Nurses over 7 years since 2015 (see Table 4). 53% of patients received more than 2 episodes of care in total over the 7 years. Patients with more care episodes (around 3–5) tended to reflect the role of community mental health nurses in providing longer-term care and monitoring.

**Table 3:** Interventions recorded by Community Mental Health Nurses.

Psychoeducation / Advice:	291
Self-help (Guided and non-guided):	198
Collaborative case management*:	639
Referral to other services:	223
Other / not recorded:	68
* Interventions delivered in Collaborative Case Management –	
Cognitive behavioural therapy	
Dialectical behaviour therapy	
Counselling	
Solution focused therapy	
Behavioural activation / activity scheduling	
Problem solving	
Behavioural Family Therapy	
Medication management	
Liaison with other services	

### 3.3 Attendance and engagement

89% of 1582 referrals attended a first appointment with a mental health nurse. 25% of patients dropped out after attending a first treatment appointment (357). 83% of 10,683 routine treatment appointments were attended. Of the 17% not attended, 9% were cancelled/rearranged at short notice and 8% did not attend without notice.

### 3.4 Outcomes from treatment

Mean self-rated pre-treatment scores for mood and anxiety were in the moderately severe, and severe ranges respectively. Mean pre-treatment ratings of impact on functioning were severe across a range of activities/areas (see Table 4). Average time from pre to post treatment scores was 17 weeks (range 4 – 41 weeks). Mean scores of both symptom severity and functioning improved significantly by the end of treatment, with large effect sizes ranging from 0.5 to 0.8, demonstrating meaningful clinical effectiveness. 4% of patients moved off psychotropic medication (accounting for those who also started medication during the study period). 67% of patients in employment returned to work from statutory sick pay, demonstrating the role of the nurses in helping people remain in employment. There was a net return to work of 5% of unemployed patients by the end of treatment, which was significant given the high levels of unemployment and economic/social deprivation across the locality.

The Wellbeing Nurses dealt with 1544 patients in primary care, of which 1418 (92%) remained with the primary care mental health service throughout treatment, and only 126 were ‘stepped up’ to secondary care mental health teams. Further to this, 249 patients were originally ‘stepped down’ to the Wellbeing Nurses from secondary care mental health services (a net movement of 123 patients from secondary to primary care mental health services).

**Table 4:** Mean pre and post-treatment scores for patients completing a course of treatment with primary care community mental health nurses (*N* = 984).

Measure pre-treatment (SD) Work and social adjustment scale items -		Post-treatment (SD)	Effect size*	Pre-post p
Work	4.8 (2.5)	3.0 (2.4)	0.7	<0.001
Home management	4.1 (2.4)	2.9 (2.2)	0.5	<0.001
Social leisure activities	4.8 (2.4)	3.2 (2.5)	0.6	<0.001
Private leisure activities	3.9 (2.5)	2.6 (2.3)	0.5	<0.001
Relationships	4.7 (2.4)	3.0 (2.3)	0.7	<0.001
PHQ-9	17.0 (6.2)	11.8 (8.3)	0.8	<0.0001
GAD-7	14.2 (5.4)	9.6 (6.8)	0.8	<0.0001
Number using medication (*N* = 1059)	425 (40%)	381 (36%)		
Number (%) moved off sick pay (Net total)	65 (67%)			
Number unemployed returned to work	32 (5%)			
at discharge sessio				

⁎ Formula: “pre-treatment mean – post-treatment mean)/pre-treatment SD; 0.8 upwards is usually regarded as large & clinically significant.

## 4 Discussion

Evidence for the effectiveness of community mental health nurses, and for specific models of primary care mental health integration has been lacking. This study demonstrates an example of the effective implementation of both. Different provider organisations collaborated in a partnership to provide a single point of access and assessment for all mental health problems in primary care. Community mental health nurses were able to collaborate with other professionals in primary care and “Step-up” patients with more complex/severe problems to the secondary care psychiatrist-led community mental health team.

### 4.1 Limitations

In this uncontrolled observational study, conclusions cannot be made on the extent to which community mental health nurses improved patient outcomes beyond usual GP care. There are also limitations to the general assumptions that can be drawn from observing the outcomes in one specific service/locality. There may be factors specific to local service structures/pathways, workforce or population that could produce results particular to this service, which may not be observed elsewhere. One such potential bias arises from the fact that a small proportion of patients did not have access to the online self-report clinical measures and had to read out their scores to their nurse at the start of appointments. This may have introduced an observer bias that could influence patients to report a more favourable score. Despite these potential biases, the observation of data collected in routine clinical practice is also a strength of the study, as it enable insight into how interventions work in real-life clinical practice, rather than selected samples under artificially controlled conditions. Such real-world outcome data has been lacking for community mental health nursing.

One of the aims of this study – to identify and examine the specific psychological interventions delivered by community mental health nurses and their effectiveness – was hampered by the clinical IT system, which did not enable nurses to record these specific interventions in the main appointment field. Nurses could only choose from a list of more general interventions such as, “Collaborative case management”. Such restrictions on routine clinical nursing records is a significant barrier to defining and understanding the specific interventions delivered by mental health nurses, and may reflect a lack of understanding/regard for the profession as providers of psychological therapies.

During the study period (2019–2022) the Covid-19 pandemic restrictions meant that from March 2020 the mental health nurses treatment sessions moved from face-to-face appointments to web-camera and telephone delivery. As restrictions lifted in 2021, treatment delivery moved to a mix of mostly web-camera and face-to-face appointments. Although these challenges were experienced by most mental health services, it is important to acknowledge that such major changes to treatment delivery may have affected the observed outcomes. Despite this, the nurses demonstrated flexibility in adapting their skills for remote delivery, whilst still recording clinically significant improvement for patients.

In an effort to reduce bias in treatment effects arising from missing outcome data, all patients with outcome measures rated on more than one occasion were included in the pre-post treatment outcome analysis, regardless of whether they dropped out of treatment. No second set of outcome ratings were recorded for 142 patients who were discharged after attending treatment sessions beyond assessment, and so they could not be included in the analysis. Problems with data quality were also identified in 73 cases, as no discharge was recorded despite having no clinical contacts for several months. These cases were therefore recorded as treatment ‘drop-outs’. 24 out of the 73 ‘drop-outs’ had multiple outcome ratings and were still included in the analysis. Outcome data was therefore missing for 16% of the 1175 patients who attended a further treatment appointment beyond assessment. Whilst such issues are often present in ‘real-world’ routine service data, missing data is always a potential source of bias, as the true outcome is unknown. Therefore, an element of caution should be employed when drawing conclusions from the presented outcomes.

### 4.2 Influential factors for effective integration

A crucial factor for integration was the shared IT system, particularly the shared electronic appointment diaries for assessments and first treatments, which ensured a co-ordinated patient journey through treatment. However, to achieve this the community mental health nurses employed directly in GP practices had to use both the shared IT system, and the GP practice IT system for their clinical notes, resulting in multiple log-ins. For this reason, there was some initial resistance from these nurses employed in GP Practices on being trained to use the shared system.

Similarly, nurses in the psychiatrist-led community mental health team of the secondary care mental health trust used a different IT system, so all primary care mental health nurses needed additional accounts on the Trust's IT system to transition patients to/from the psychiatrist-led service. Linked IT systems have been identified as an important factor in the successful coordination of integrated care (Kozlowska et al., 2018), and a review by Monitor (2012) in the UK also identified the quality of IT and communication systems as a significant obstacle to primary and secondary care integration.

Yet despite multiple IT systems and providers, the service was still able to achieve integrated working. The main reason for this was co-ordinated training sessions for all the mental health professionals on both the different clinical IT systems, and each other's roles, to inform decisions on suitability. Training for each mental health professional took three days but led to a greater understanding of each other's roles and working practices. During training the nurses fed-back suggestions to the Clinical Lead on aspects of service design. This gave them some ownership and control over the new and unfamiliar systems/processes. Such shared responsibility can help foster mutual trust and overcome initial resistance to sharing clinical appointment diaries, which can be opened-up to be booked into by others. This aspect of the service was arguably the most important in achieving a single assessment in primary care. Nurses became more willing to use the multiple IT systems for different tasks once they experienced the satisfaction of improved patient care.

### 4.3 Effectiveness of community mental health nurses in primary care

Community mental health nurses delivering psychological interventions in primary care demonstrated clinically meaningful improvement in both symptoms and functioning of patients with a wide range of mental health problems. The nurses reduced the burden on secondary care mental health services and helped people remain in work – even demonstrating a 5% net return to employment for patients by the end of treatment/follow-up.

Whilst this was a service evaluation rather than a controlled study, the large pre-post treatment effect sizes of 0.8 for both depression (PHQ-9) and anxiety (GAD-7) are larger than those reported in a similar uncontrolled evaluation of a psychological therapy service for common mental health problems (PHQ-9: 0.6; GAD-7: 0.6, Seaton et al., 2022), and also larger than those in a controlled study of the long-term outcomes of psychological therapies for depression and anxiety (PHQ-9: 0.6; GAD-7: 0.5, Solbjørg et al., 2020). Patients treated by the nurses in this study did not have anxiety disorders and/or depression as their main problem, so these symptoms may have been more amenable to change. However, the observed effect sizes of 0.8 were also greater than those of 0.3 achieved in two previous studies of community mental health nursing that delivered generic interventions for all mental health problems, such as Problem-Solving (Kendrick et al., 2005) and Interpersonal Community Psychiatric Treatment (Veen et al., 2021). An important difference in this study was that the community mental health nurses employed a range of specific evidence-based psychological treatments that were targeted for specific disorders in line with evidence-based guidance (e.g. Dialectical Behaviour Therapy for emotional dysregulation; Cognitive Behavioural Interventions for Eating Disorders). The role of the community mental health nurses was therefore closer to that of psychological therapists delivering problem-focused treatment programmes, with some patients receiving longer-term nursing care.

Engagement with mental health nurses was good, as only 11% of referrals failed to attend a first appointment. This is significantly better than the national did not attend (DNA) rate of psychiatric outpatient appointments in England, which was 19.1% in 2021–22 (Department of Health 2022). Once patients entered treatment, non-attendance rates for weekly appointments rates rose to 17%. The service target for mental health nurses to spend over 50% of their time with patients was aided by the shared electronic appointment diary system, which enabled staff to book patients into cancelled appointments with any nurse. This increased the efficiency of nursing care.

The nurses worked alongside physical healthcare professionals, such as GPs and Practice Nurses, who advised patients to self-refer to the Wellbeing Service. 37% of patients treated by Wellbeing Nurses had a co-morbid long term physical health condition, and 17% had a registered disability, which is higher than the national estimate of 30% (Naylor et al., 2012). This indicates effective pathways between physical and mental health professionals in primary care, even though only 13% of referrals to the nurses came directly from physical health teams. Several studies have demonstrated that effective treatment for mental health problems reduces unscheduled appointments/admissions for co-morbid physical health problems (NHS England, 2018; Howard et al., 2010; Moore et al., 2007). Therefore, the integration of mental health nurses into primary care is also likely to have helped ease pressures on local physical healthcare teams.

Therapists in the ‘Improving access to psychological therapies’ programme (who specialised in treating common mental health problems) booked 307 patients directly into first appointments with the community mental health nurses. This was mainly due to problems such as emotional dysregulation and self-harm preventing patients from engaging with structured therapies such as CBT. Indicators of personality disorder are predictors of poorer outcome from ‘Improving access to psychological therapy’ services, as therapists are only trained to treat anxiety and depression (Goddard et al., 2015). Conversely, the community mental health nurses also booked 267 patients directly into first appointments with these therapists. During the 3-year study period the ‘Improving access to psychological therapies’ team demonstrated an average recovery rate of 56% (consistently higher than the national average of 51%, NHS Digital, 2021). Integrating community mental health nurses with these therapists may have therefore helped to improve their recovery rates by ensuring they focused on treating anxiety disorders and depression rather than other problems.

### 4.4 Implications for service design, practice and research

This study demonstrates the utility of an integrated model of treatment delivery by primary care mental health nurses alongside psychological therapists and physical health professionals. The nurses delivered a “Stepped-care” model of treatment, in which the intensity of treatment provision is increased/decreased in line with severity/outcomes (see ‘The Service’). Such a model is well suited to caring for people with emotional dysregulation, as it prevents the transitions between services that can increase risks in this group of patients (NICE, 2015). Such stepped-care principles could be explored and tested across different configurations of integrated services.

The primary care mental health nurses delivered a range of psychological therapies for specific problems in line with evidence-based guidance. Mental health nurses are routinely trained to, “Use a range of evidence-based psychological, psychosocial and other complex therapeutic skills” (HEE, 2020), including the provision of evidence-based therapeutic interventions, such as: Talking Therapies; Cognitive Behavioural Therapy techniques; Solution-Focused therapies; Motivational Interview techniques and Positive Behavioural Approaches (NMC, 2018). Yet Mental Health Nurses in the UK are not recognised as members of the psychological professions workforce in national expansion and training plans (Health Education England, 2021). Given the lack of published studies on the effectiveness of mental health nurses in delivering psychological interventions, there is a need for more research and guidance on this aspect of the role, which is central to integrated primary care mental health teams.

The clinical IT system used by mental health nurses did not include any psychological therapies in the choice of primary interventions they were required to record at each appointment. This differed to the choices presented to the therapists in the ‘Improving access to psychological therapies’ programme, who could choose from a full range of therapies. If research into the provision of psychological therapies is to include mental health nurses, these interventions need to be included in the clinical IT systems used to report routine nursing practice.

Further research is required on the impact of treatment by primary care mental health nurses on subsequent physical healthcare utilisation and treatment for co-morbid long term physical conditions (LTCs) in primary care. This should also include the effect of co-morbid LTCs on outcomes for mental health problems.

## 5 Conclusion

Primary care community mental health nurses successfully integrated with other mental health professionals to form a single point of access for mental health problems in primary care. Delivered across general primary care sites, the “Wellbeing Service” routinely addressed the mental health of patients alongside their physical healthcare. The community mental health nurses delivered a stepped care model of treatment and were partially integrated with secondary care community mental health teams to seamlessly ‘step-up/down’ patients when required. Nurses shared knowledge and understanding of their different roles/procedures, and shared IT systems/diaries allowed coordinated care within a centralised system of clinical governance. Primary care community mental health nurses were clinically effective in delivering psychological therapies, targeted for specific problems in line with evidence-based guidance.

## Funding sources

No external funding.

## CRediT authorship contribution statement

**Mark Kenwright:** Writing – original draft, Formal analysis, Conceptualization. **Paula Fairclough:** Project administration, Data curation, Conceptualization. **Jason McDonald:** Writing – review & editing, Methodology, Investigation. **Louisa Pickford:** Writing – review & editing, Methodology, Investigation.

## Declaration of competing interest

None.

## Data Availability

Data not available / The data that has been used is confidential:.
